# Network Protein Interaction in the Link between Stroke and Periodontitis Interplay: A Pilot Bioinformatic Analysis

**DOI:** 10.3390/genes12050787

**Published:** 2021-05-20

**Authors:** Yago Leira, Paulo Mascarenhas, Juan Blanco, Tomás Sobrino, José João Mendes, Vanessa Machado, João Botelho

**Affiliations:** 1 Periodontology Unit, Faculty of Odontology and Medicine, Medical-Surgical Research Group, Health Research Institute of Santiago de Compostela, University of Santiago de Compostela, 15706 Santiago de Compostela, Spain; yagoleira@gmail.com (Y.L.); jblanco@blancoramos.net (J.B.); 2 Clinical Neurosciences Research Laboratory, Health Research Institute of Santiago de Compostela, University Clinical Hospital, 15706 Santiago de Compostela, Spain; Tomas.Sobrino.Moreiras@sergas.es; 3 Periodontology Unit, UCL Eastman Dental Institute & NIHR UCLH Biomedical Research Centre, University College London, London WC1E 6BT, UK; 4 Center for Medical Genetics and Pediatric Nutrition Egas Moniz, Instituto Universitário Egas Moniz (IUEM), 2829-511 Caparica, Portugal; pmascarenhas@egasmoniz.edu.pt; 5 Evidence-Based Hub, Clinical Research Unit (CRU), Centro de Investigação Interdisciplinar Egas Moniz (CiiEM), Egas Moniz—Cooperativa de Ensino Superior, CRL, 2829-511 Caparica, Portugal; jmendes@egasmoniz.edu.pt (J.J.M.); vmachado@egasmoniz.edu.pt (V.M.); 6 Periodontology Department, Clinical Research Unit (CRU), Centro de Investigação Interdisciplinar Egas Moniz (CiiEM), Egas Moniz—Cooperata de Ensino Superior, CRL, 2829-511 Caparica, Portugal

**Keywords:** stroke, periodontitis, periodontal disease, protein–protein network interaction, bioinformatics

## Abstract

The clinical interaction between stroke and periodontitis has been consistently studied and confirmed. Hence, exploring potentially new protein interactions in this association using bioinformatic strategies presents potential interest. In this exploratory study, we conducted a protein–protein network interaction (PPI) search with documented encoded proteins for both stroke and periodontitis. Genes of interest were collected via GWAS database. The STRING database was used to predict the PPI networks, first in a sensitivity purpose (confidence cut-off of 0.7), and then with a highest confidence cut-off (0.9). Genes over-representation was inspected in the final network. As a result, we foresee a prospective protein network of interaction between stroke and periodontitis. Inflammation, pro-coagulant/pro-thrombotic state and, ultimately, atheroma plaque rupture is the main biological mechanism derived from the network. These pilot results may pave the way to future molecular and therapeutic studies to further comprehend the mechanisms between these two conditions.

## 1. Introduction

Stroke is a highly prevalent neurological disease affecting one in four adults worldwide [1,2]. It is the second leading cause of death (accounting for almost 6 million deaths/year) and the third leading cause of disability in the world [1,2]. Most stroke cases are ischemic due to reduced blood flow caused by arterial occlusion. The remaining stroke presentations are haemorrhagic, resulting from rupture of cerebral arteries [3]. A recent economic analysis in Europe reported that stroke cost EUR 60 billion, with health care accounting for EUR 27 billion (45%), representing 1.7% of health expenditure [4].

Periodontitis, a chronic oral inflammatory and infectious disease that is characterized by destruction of soft and hard gingival tissues, affects more than half of adults [5]. It has been estimated that the most advanced forms of this disease affect around 11% of the adult population worldwide [6]. More importantly, periodontitis not only affects the gingival tissues but also contributes to the body’s overall inflammatory burden, which makes this disease a potential risk factor for atherosclerotic inflammatory vascular diseases such as stroke [7].

A link between periodontitis and stroke, mainly ischemic stroke, has been established over the last few decades [8]. Moreover, stroke survivors with periodontitis are more prone to have worse prognosis and more likely to suffer recurrent vascular events than those without periodontitis [9,10]. Potential mechanisms proposed behind the association between periodontitis and ischemic stroke might include activation of innate immune system, systemic inflammation leading to vascular endothelial dysfunction, increased cholesterol biosynthesis, and prothrombotic state. All of them may exacerbate atherosclerotic lesions and increase the risk of atherosclerosis, thus promoting cerebrovascular events including large and small vessel cerebral infarcts [11]. However, a recent Mendelian randomization study failed to demonstrate a causal genetic association between both diseases where only five single nucleotide polymorphisms were used from a number of genome-wide association studies [12].

To better understand the contribution of genetics to this relationship, genetic risk factors should be analysed and integrated in terms of biological pathways and functions [13]. For this purpose, protein interaction data, derived from a wide range of cellular and biochemical model systems, can be used for building protein–protein interaction (PPI) networks from genes associated with both periodontitis and stroke. PPI network analysis could be, therefore, a powerful time- and cost-effective approach to identify potential biological pathways, key players, or candidate genes involved in the periodontitis-stroke link.

Hence, the aim of this study was to identify the key proteins and the biological regulatory pathways involved in both periodontitis and stroke physiopathology.

## 2. Materials and Methods

### 2.1. Data Source

The National Human Genome Research Institute-European Bioinformatics Institute Catalog of Human Genome-Wide Association Studies (NHGRI-GWAS) was used to search potential GWAS datasets [14]. The NHGRI-GWAS is an all-inclusive catalogue with publicly available summary statistics, facilitating access and replicability. Periodontitis GWAS studies were used accounting for up to 100,903 individuals of European, Asian, American and other ancestries [15,16,17,18,19,20,21,22,23,24,25,26,27] (Supplementary S1), as in [28]. In this, we included the link where genes were reported in the GWAS search [29].

For stroke, we used summary GWAS statistics performed in over 2,000,000 individuals of European, Asian, American, Sub-Saharan African, Caribbean and other ancestries [30,31,32,33,34,35,36,37,38,39,40,41,42,43,44,45,46,47,48,49,50,51,52,53,54,55,56,57,58,59,60,61,62] (Supplementary S2). In this, we included the link where genes were reported in the GWAS search [63]. Both GWAS data for stroke and periodontitis were resulting from different populations as none of the included studies had data combining both conditions.

### 2.2. Protein–Protein Interaction Networks Functional Enrichment Analysis

To forecast potential PPI networks, we used the STRING (Search Tool for the Retrieval of Interacting Genes/Proteins) database, through heuristic methods of association and analysis. This platform renders possible protein networks of interaction via high-throughput experimental data, literature, and predictions grounded on genomic context analysis [64,65]. Five main sources contribute to this bioinformatic output: Genomic Context Predictions, High-throughput Lab Experiments, (Conserved) Co-Expression, Automated Textmining and Previous Knowledge in Databases. The Universal Protein Resource provided the characteristics of all proteins [66]. Then, the STRING tool provides a node–node interaction score calculated as detailed in [67].

### 2.3. Data Management, Test Methods and Analysis

After download the GWAS datasets of periodontitis and stroke, data was handled through Microsoft Office Excel. Then, we used the ‘Multiple protein’ to render PPI networks at STRING version 10.5. We carried a first analysis with a confidence cut-off of 0.7 to serve as sensitivity analysis aiming to infer the dependency on the choice of the confidence cut-off. Then, we defined a cut-off of 0.9, the highest confidence in the final interaction examination. In the resultant PPI network, proteins are presented as nodes related through lines, and nodes thickness increase reveals higher confidence level. Furthermore, ‘ggplot’ for R was used to assemble a gene interaction heatmap.

### 2.4. Gene Enrichment Analysis

Then, to investigate whether there were genes over-represented in the final network and its possible effect, we run the list of proteins in The Geneontology Resource (http://geneontology.org/ accessed on February 2021). A ‘Reactome pathways’ was executed, and false discovery rates were computed using Fisher’s Exact. Functional enrichment analysis was further confirmed using the WebGestalt tool (http://www.webgestalt.org/ accessed on April 2021).

## 3. Results

### 3.1. Protein–Protein Interaction Analysis

**Figure 1:**
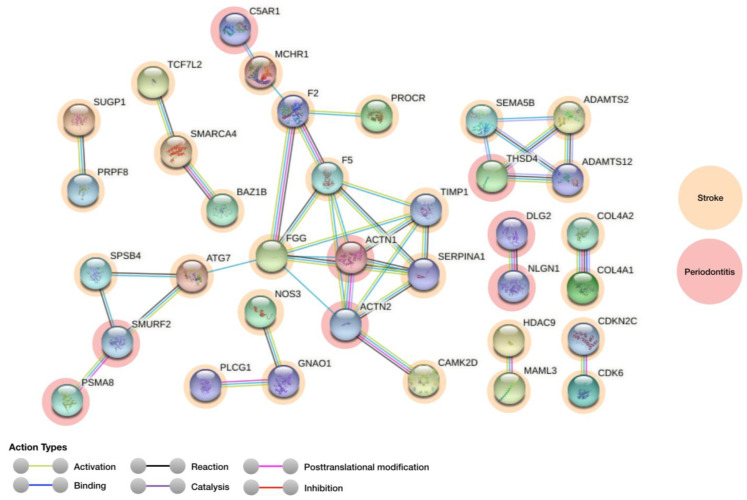
PPI network between stroke and periodontitis relevant proteins at the highest confidence cut-off of 0.9 in this network. Mapped genes for stroke and periodontitis are coloured in orange and red, respectively.

According to the STRING results, a sensitivity analysis with a confidence cut-off of 0.7 (Supplementary S3) exposed dependency on the confidence cut-off, as a more concise network emerged with a confidence cut-off of 0.9 (Figure 1). Overall, 148 nodes with 41 PPI relationships (from 32 expected edges) were found (Figure 1). The PPI enrichment significance was 0.0795, indicating the current set of proteins is small and the result of these proteins have not been studied very much. The average node degree was 0.554, and the average local average local clustering coefficient was of 0.194.

**Figure 2:**
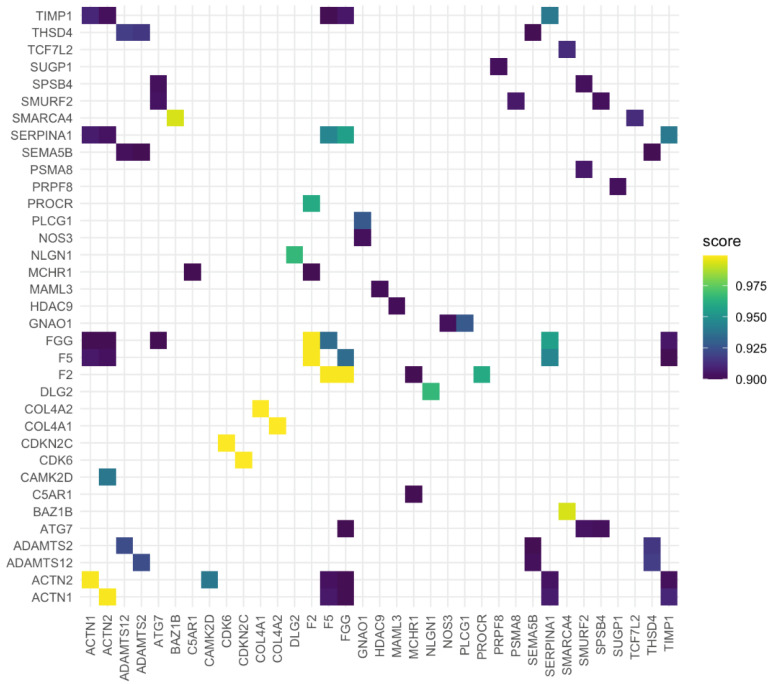
Heatmap towards the confirmation of gene interaction. Both axis represent the list of genes of interest. *ACTN1*—Actinin α 1; *ACTN2*—Actinin α 2; *ADAMTS12*—ADAM metallopeptidase with thrombospondin type 1 motif 12; *ADAMTS2*—ADAM metallopeptidase with thrombospondin type 1 motif 2; *ATG7*—Autophagy related 7; *C5AR1*—Complement C5a receptor 1; *CAMK2D*—Calcium/calmodulin dependent protein kinase II delta; *F2*—Coagulation factor II, thrombin; *F5*—Coagulation factor V; *FGG*—Fibrinogen γ chain; *MCHR1*—Melanin concentrating hormone receptor 1; *SEMA5B*—Semaphorin 5B; *SERPINA1*—Serpin family A member 1; *SMURF2*—E3 ubiquitin–protein ligase SMURF2; *SPSB4*—SplA/ryanodine receptor domain and SOCS box containing 4; *THSD4*—Thrombospondin type 1 domain containing 4; *TIMP1*—TIMP metallopeptidase inhibitor 1.

The casted network display possible PPIs between known associated genes stroke and periodontitis (Figure 1, Table 1). Furthermore, the physiological characteristics and localization of each encoded protein is presented in Table 2. The gene interaction was further confirmed via heatmap plot (Figure 2) and Genemania network corroborated a PPI similar network obtained in STRING database (Supplementary S4).

### 3.2. Gene Enrichment Assessment and Gene Ontology

The over-representation analysis with “Reactome pathways” revealed some over-representation in terms of: fibrin clot formation; CREB phosphorylation through the activation of CaMKII; Ras activation upon Ca^2+^ influx through NMDA receptor; unblocking of NMDA receptors, glutamate binding and activation; defective B3GALTL causes Peters-plus syndrome (PpS); O-glycosylation of TSR domain-containing proteins; Platelet degranulation; Platelet activation, signalling and aggregation; haemostasis (Supplementary S5). The WebGestalt tool confirmed the gene enrichment assessment performed.

Furthermore, upon the genet ontology of these significant genes, the most significant molecular functions were: titin binding (strength = 2.22; false discovery rate [FDR] = 0.0057); nuclear receptor transcription coactivator activity (strength = 1.65; FDR = 0.0192); ion channel binding (strength = 1.46; FDR = 0.0070); cell adhesion molecule binding (strength = 1.24; FDR = 0.0153); peptidase activity (strength = 0.86; FDR = 0.0313); protein homodimerization activity (strength = 0.84; FDR = 0.0153); signaling receptor binding (strength = 0.78; FDR = 0.0023); identical protein binding (strength = 0.66; FDR = 0.0138); protein binding (strength = 0.32; FDR = 0.0323).

Concerning cellular components, the most significant components were: pseudopodium (strength = 2.13; FDR = 0.0012); platelet α granule lumen (strength = 2.01; FDR = 4.0 × 10^−9^); endoplasmic reticulum-Golgi intermediate (strength = 1.54; FDR = 0.0122); COPII-coated to Golgi transport vesicle (strength = 1.49; FDR = 0.0148); secretory granule lumen (strength = 1.4; FDR = 2.86 × 10^−7^).

## 4. Discussion

In this exploratory bioinformatic analysis, a likely PPI network between stroke and periodontitis was foreseen using open-access catalogues of GWAS studies in humans. This forecasted PPI network needs careful interpretation and warrants preclinical and clinical validations, nevertheless shows new evidence that will empower new studies on the association between stroke and periodontitis. Below we present different hypothetical biological scenarios based on our PPI network through which periodontitis can increased the risk of stroke.

The largest node found with the PPI network is related to the atherosclerotic process. In the early stages of atherogenesis, two α actinins associated with periodontitis, namely *ACTN-1* and *-2*, can bind to intracellular adhesion molecule 1 (*ICAM-1*) providing a “firm foothold” for leukocytes undergoing migration between endothelial cells into the intima layer of the artery and the underlying inflammatory lesion [65]. In this phase, endothelial dysfunction can be seen as the endothelial lining partially has lost its integrity. In this sense, *ACTN-1* and *-2* interact with *SERPINA1*, which encodes α 1-antitrypsin (*A1AT*), a protease inhibitor that protects surrounding tissues at sites with inflammation and has been found in human atherosclerotic lesions [66]. On the other hand, after pro-inflammatory cytokines and matrix metalloproteinases (MMPs) and its tissue inhibitor (e.g., *TIMP-1*) upregulation takes place due to lipid streaks and calcification, the atheroma plaque starts the maturation process. This process together with development of a compensatory blood supply within the atherosclerotic lesion, will lead to additional activation and proliferation of pro-inflammatory cells and production of thrombin (interaction *TIMP-1* with *F5*). In this stage, the endothelial lining is all lost and fibrinogen is enzymatically generated from fibrin. This clotting cascade (interaction *F2* and *F5* with *FGG*) will result in formation of thrombus (i.e., thrombosis) and atherosclerotic cerebrovascular disease will develop latter on. MMPs overexpression leads to degradation of collagen, weakening of the vessel’s strength and fissures in the atheroma (interaction between TIMP-1 with *SERPINA1*). In some cases, where atheroma keeps progressing, a large necrotic core is exposed to the vasculature with the lesion which results in contact with platelets, coagulation is initiated and ultimately plaque rupture (i.e., vulnerable lesions) (interaction between *FGG* and *SERPINA1*).

In the interrelation of pro-coagulant state and formation of thrombus, another interesting node was observed in the PPI network mainly consisted of metallopeptidase with thrombospondin type 1 motif 2 and 12 (*ADAMTS2* and *12*) and Thrombospondin type 1 domain containing 4 (*TSH4*). In addition, they are produced by vascular smooth muscle cells and platelets, *ADAMTS* have also a key role in maintaining cardiovascular haemostasis and down-regulating coagulation by inhibition of thrombin [67]. For example, an increase in *ADAMTS12* activity would hypothetically lead to less inhibition of thrombin and an elevated risk for ischemic stroke. On the other hand, genetic variations of the *ADAMTS* family such as *ADAMTS2* and 12 have shown to reduce the integrity of the endothelial lining, which together with inflammatory processes and defective vascular remodelling might play a key role in cerebral aneurysms pathogenesis [68]. Thrombospondin 1 is elevated in gingival tissues with periodontitis and this overexpression is induced by lipopolysaccharide from *Porphyromonas gingivalis* (a keystone pathogen) via innate immunity system activation and inflammatory responses [69]. Therefore, biologically, it would be plausible that periodontitis through the interaction of *TSH4* and *ADAMTS2* and *12* is associated with stroke and cerebral aneurysms.

Calcium/calmodulin dependent protein kinase II (*CAMK2*) has been related to ischemic neuronal death due to glutamate-mediated excitotoxicity [70]. In the present PPI network *ACTN2* (a protein which main role is to anchor actin with several intracellular molecules) was associated with *CAMK2D*, which is involved in the regulation of Ca^2+^ homeostasis and excitation-contraction. Concentrations of some amino acids such as arginine and glutamate were altered in gingival crevicular fluid of periodontal patients [71]. Therefore, the pathway *ACTN2-CAMK2* deserved further investigation, as it could be an interesting and relevant biological mechanism behind the relationship between periodontal destruction and cerebral ischemia.

Other associations derived from the present PPI network includes complement system activation (*C5AR1*) and increased pro-coagulant/thrombotic (*F2*) state via melanin. Increased local activation of complement products such as C5 in the periodontal tissues increases the intensity of the local inflammatory response, resulting in enhanced vascular permeability and vasodilatation and recruitment of inflammatory cells, which, in turn, will lead to excessive release of reactive oxygen species and interleukins which promotes accumulation of immune cells [72]. Hence, a potential link between activation of immune systemic via C5 activation and overproduction of for instance fibrinogen, a molecule related to systemic inflammation and intimately involved in the development of atherosclerosis could be feasible. Research needs to be done investigating the mechanism underlying this finding as well as to study the exact role of melanin in this link.

The last node to be highlighted is the one which relates ubiquitination, autophagy and coagulation. For instance, in our PPI network we found that Smad ubiquitination regulatory factor-2 (*SMURF2*), an ubiquitin E3 ligase responsible for proteasome-mediated degradation of enhancer of zeste homolog 2 *EZH2* which is a process required for neuron differentiation [73] is present in periodontitis. This ubiquitin was associated with the process of autophagy in stroke and ultimately with promotion of coagulation. Again, a potential biological pathway has been described for the perio–stroke relationship.

Importantly, we highlight that all these proposed interactions are hypothetical and demand further validation. Furthermore, the inflammatory machinery is inherent in the vast majority of diseases and is still far from being fully understood and, therefore, is very difficult to be established as a definitive criterion of interaction between these two conditions. Thus, the reader must bear in mind that the aforementioned lines of interaction are, at this stage, contingent on further evidence.

Overall, this exploratory report foresees a comprehensive analysis using free and large outputs. However, a number of shortcomings are relevant to discuss. First, this sort of analysis is dependent on the number of genes included in GWAS, and future studies with a higher number of genes disclosed will result in new paths of interaction. Second, network protein interaction approaches have limited clinical predictive value [68], but unveil hypothetical new paths of interaction. On the other hand, the quantity of SNPs of interest in these datasets has combined a diversity of ethnical background allowing the generalization of our results. Beyond these limitations, our sample size (over 2 million people) makes the results persuasive. Furthermore, we have also provided a protein-enrichment analysis, to overlook the over-representation level e in the observed PPI.

## 5. Conclusions

This exploratory bioinformatic analysis shows a potential network of interaction between proteins related to stroke and periodontitis, respectively. The main biological pathway identified relies on inflammatory response, pro-coagulant/pro-thrombotic state and subsequent mechanisms of atheroma plaque rupture. These results may shed light on future molecular and therapeutic studies to further grasp the association between these two conditions.

## Figures and Tables

**Table 1:** Interaction weight between stroke and periodontitis genes identified in the network.

Genes for Stroke (Regulation)	Genes for Periodontitis (Regulation)	Score
*CAMK2D*	*ACTN2*	0.939
*ADAMTS12*	*THSD4*	0.918
*ADAMTS2*	*THSD4*	0.916
*SERPINA1*	*ACTN1*	0.907
*F5*	*ACTN1*	0.906
*ATG7*	*SMURF2*	0.904
*SERPINA1*	*ACTN2*	0.904
*F5*	*ACTN2*	0.903
*SPSB4*	*SMURF2*	0.902
*MCHR1*	*C5AR1*	0.900
*SEMA5B*	*THSD4*	0.900

**Table 2:** Details of the identified genes in the interaction between stroke and periodontitis.

Gene Symbol	Name	Description	Localization
**Stroke**			
*F2*	Coagulation factor II, thrombin	Cleaves bonds after Arg and Lys, converts fibrinogen to fibrin and activates factors V, VII, VIII, XIII, and, in complex with thrombomodulin, protein C. Functions in blood homeostasis, inflammation and wound healing	Plasma and Liver
*F5*	Coagulation factor V	Regulator of hemostasis. Is a critical cofactor for the prothrombinase activity of factor Xa that results in the activation of prothrombin to thrombin	Golgi apparatus
*SERPINA1*	Serpin family A member 1	Inhibitor of serine proteases	Vesicles
*CAMK2D*	Calcium/calmodulin dependent protein kinase II delta	Involved in the regulation of Ca^2+^ homeostasis and excitation-contraction	Plasma membrane, cytosol, cell junctions
*ADAMTS2*	ADAM metallopeptidase with thrombospondin type 1 motif 2	Cleaves the propeptides of type I and II collagen prior to fibril assembly (By similarity)	Plasma membrane, vesicles
*ADAMTS12*	ADAM metallopeptidase with thrombospondin type 1 motif 12	Metalloprotease that may play a role in the degradation of COMP. Cleaves also α-2 macroglobulin and aggregan. Has anti-tumorigenic properties	Nucleoli and mitochondria
*ATG7*	Autophagy related 7	Involved in the 2 ubiquitin-like systems required for cytoplasm to vacuole transport (Cvt) and autophagy	Cytosol, Plasma membrane, Nucleoplasm
*SPSB4*	SplA/ryanodine receptor domain and SOCS box containing 4	Mediates the ubiquitination and subsequent proteasomal degradation of target proteins	Nucleoplasm and Golgi apparatus
*MCHR1*	Melanin concentrating hormone receptor 1	Receptor for melanin-concentrating hormone	Not available
*FGG*	Fibrinogen γ chain	With fibrinogen α (FGA) and fibrinogen β (FGB), polymerizes to form an insoluble fibrin matrix. Has a major function in haemostasis as one of the primary components of blood clots	Endoplasmic Reticulum
*SEMA5B*	Semaphorin 5B	Acts as positive axonal guidance cues	Cytosol
***Periodontitis***			
*TIMP1*	TIMP metallopeptidase inhibitor 1	Growth factor, Metalloenzyme inhibitor, Metalloprotease inhibitor, Protease inhibitor	Golgi apparatus
*ACTN1*	Actinin α 1	F-actin cross-linking protein which is thought to anchor actin to a variety of intracellular structures	Actin filaments
*ACTN2*	Actinin α 2	F-actin cross-linking protein which is thought to anchor actin to a variety of intracellular structures	Actin filaments
*THSD4*	Thrombospondin type 1 domain containing 4	Promotes FBN1 matrix assembly	Extracellular matrix
*SMURF2*	E3 ubiquitin–protein ligase SMURF2	Involved in the transfer of the ubiquitin to targeted substrates. Interacts with SMAD1 and SMAD7 triggering ubiquitination and degradation.	Plasma Membrane, Nucleus Cytoplasm, Membrane Raft
*C5AR1*	Complement C5a receptor 1	Receptor for the chemotactic and inflammatory peptide anaphylatoxin C5a	Golgi apparatus and vesicles

## Data Availability

Data will be provided upon reasonable request from the corresponding author.

## Supplementary Materials

The following are available online at https://www.mdpi.com/article/10.3390/genes12050787/s1, Supplementary S1: Genes and their corresponding proteins for Periodontitis, Supplementary S2: Genes and their corresponding proteins for stroke, Supplementary S3: PPI Network with confidence cut-off of 0.7, Supplementary S4: PPI Network using Genemania tool, Supplementary S5: Enrichment analysis.
