# Supplementary material for: Network Protein Interaction in the Link between Stroke and Periodontitis Interplay: A Pilot Bioinformatic Analysis
Source: Genes (Basel). 2021 May 20;12(5):787. doi: 10.3390/genes12050787 (PMC8160956; doi:10.3390/genes12050787)
Supplement: Supplementary file 1 [file genes-12-00787-s001.zip › genes-1193465-supplementary.pdf]

Appendix S1. Genes and their corresponding proteins for Periodontitis

Mapped gene	Reported trait
GLT6D1	Periodontitis
AL360006.1, KCNK1	Periodontal microbiota
PKN2-AS1	Periodontal microbiota
CLIC5, RUNX2	Periodontal microbiota
Y_RNA, AC064802.1	Periodontal microbiota
CAMTA1	Periodontal microbiota
AF111167.2, LINC01220	Periodontal microbiota
TENM2	Periodontal microbiota
GRID1	Periodontal microbiota
AL360006.1, KCNK1	Periodontal microbiota
AC016751.2, EXTL2P1	Periodontal microbiota
DAB2IP, AL596244.1	Periodontal microbiota
Y_RNA, NAMPTP1	Periodontal microbiota
FAM166C, OTOF	Periodontal microbiota
FBXO38	Periodontal microbiota
UHRF2	Periodontal microbiota
TBC1D1	Periodontal microbiota
AC003044.1	Periodontitis
AL136967.2, FOXP4-AS1	Periodontitis
ADGRE1	Periodontitis
TTC6	Periodontitis (CDC/AAP)
GPN1	Periodontitis (CDC/AAP)
HLA-DOA	Periodontitis (CDC/AAP)
ITGA8	Periodontitis (CDC/AAP)
ERGIC1	Periodontitis (CDC/AAP)
LRP1B	Periodontitis (CDC/AAP)
PRB2, AC078950.1	Periodontitis (CDC/AAP)
C10orf91, AL451069.1	Periodontitis (CDC/AAP)
AL161449.2, Y_RNA	Periodontitis (CDC/AAP)
CEP295NL, TIMP2	Periodontitis (CDC/AAP)
ACTN2	Periodontitis (CDC/AAP)
KDM4B	Periodontitis (CDC/AAP)
LINC01828, AC007403.1	Periodontitis (CDC/AAP)
AC022239.2, LINC00208	Periodontitis (CDC/AAP)
AC015987.1	Periodontitis (CDC/AAP)
MFSD1	Periodontitis (CDC/AAP)
LINC01262, HSP90AA4P	Periodontitis (CDC/AAP)
IGF2R	Periodontitis (CDC/AAP)
WDR73, SCAND2P	Periodontitis (CDC/AAP)
PARP15	Periodontitis (CDC/AAP)
RPL35AP19, AC068413.1	Periodontitis (CDC/AAP)

CSMD1	Periodontitis (CDC/AAP)
ETNK2	Periodontitis (CDC/AAP)
AC010468.1, AC010468.3	Periodontitis (PAL4Q3)
THSD4	Periodontitis (PAL4Q3)
ROBO2	Periodontitis (PAL4Q3)
LINC01748, LINC02778	Periodontitis (PAL4Q3)
DEFA10P, DEFA9P	Periodontitis (PAL4Q3)
NKAIN3	Periodontitis (PAL4Q3)
PPIAP65, LINC01854	Periodontitis (PAL4Q3)
IGLV10-54	Periodontitis (PAL4Q3)
ROCK1P1	Periodontitis (Mean PAL)
AC055874.1	Periodontitis (Mean PAL)
SMURF2	Periodontitis (Mean PAL)
AC027229.1, RN7SL97P	Periodontitis (Mean PAL)
AC015871.1, ST20-MTHFS	Periodontitis (Mean PAL)
THSD4	Periodontitis (Mean PAL)
ZNF579	Periodontitis (Mean PAL)
ABCA1	Periodontitis (Mean PAL)
MIR8052, BAK1P2	Periodontitis (Mean PAL)
BCORL1, AL034405.1	Periodontitis (Mean PAL)
BIRC5	Periodontitis (Mean PAL)
AC063949.1, C12orf74	Periodontitis (Mean PAL)
ST20-AS1, AC015871.2	Periodontitis (Mean PAL)
CDH13, AC009063.2	Periodontitis (Mean PAL)
AC114324.2, LINC02062	Periodontitis (Mean PAL)
ICE2P2, NDUFA5P5	Periodontitis (Mean PAL)
AP001042.1	Periodontitis (Mean PAL)
AC093534.2, RPL35AP15	Periodontitis (DPAL)
DAOA-AS1, AL138954.1	Periodontitis (DPAL)
GRIK1	Periodontitis (DPAL)
AC093534.2, RPL35AP15	Periodontitis (DPAL)
NLGN1	Periodontitis (DPAL)
AC006059.2, CCDC13	Periodontitis (DPAL)
PWRN1, AC090983.2	Periodontitis (DPAL)
SUMF1	Periodontitis (DPAL)
LBP	Periodontitis (DPAL)
LINC02022	Periodontitis (DPAL)
AC005208.1, KCNJ16	Periodontitis (DPAL)
SCN2A	Periodontitis (DPAL)
TGIF1	Periodontitis (DPAL)
NPAP1, AC090983.2, PWRN1	Periodontitis (DPAL)
ZFPM2	Periodontitis (DPAL)
PPIAP65, LINC01854	Periodontitis (PAL4Q3)

LINC00907	Periodontitis (PAL4Q3)
RBFOX1	Periodontitis (PAL4Q3)
LINC01933	Periodontitis (PAL4Q3)
HMGB1P5, AC092421.1	Periodontitis (PAL4Q3)
DEFA10P, DEFA9P	Periodontitis (PAL4Q3)
LINC00907	Periodontitis (PAL4Q3)
THSD4	Periodontitis (PAL4Q3)
FAM135B	Periodontitis (PAL4Q3)
AC010468.1, CAMK4	Periodontitis (PAL4Q3)
FAM126A	Periodontitis (Mean PAL)
NKAIN2	Periodontitis (Mean PAL)
PSMA8	Periodontitis (Mean PAL)
ERC2	Periodontitis (Mean PAL)
MIR8052, BAK1P2	Periodontitis (Mean PAL)
ACTN1	Periodontitis (Mean PAL)
DKK1, RPL31P44	Periodontitis (Mean PAL)
AL591501.1	Periodontitis (Mean PAL)
FIZ1	Periodontitis (Mean PAL)
HMX3, ACADSB	Periodontitis (Mean PAL)
HS6ST2	Periodontitis (Mean PAL)
LINC01278	Periodontitis (Mean PAL)
AC090666.1, LINC01919	Periodontitis (Mean PAL)
NKAIN2	Periodontitis (Mean PAL)
HNRNPA1P58, NRSN1	Periodontitis (Mean PAL)
AC123023.1, LINC01811	Periodontitis (Mean PAL)
GDF15	Periodontitis (Mean PAL)
NPM1P2	Periodontitis (Mean PAL)
AC063965.1, PTEN	Periodontitis (Mean PAL)
PTPRT	Periodontitis (Mean PAL)
CSMD1	Periodontitis (CDC/AAP)
RGMA, AC108457.1	Periodontitis (CDC/AAP)
AC022239.2, LINC00208	Periodontitis (CDC/AAP)
AC022239.2, LINC00208	Periodontitis (CDC/AAP)
KDM4B	Periodontitis (CDC/AAP)
AC090365.1, CDH2	Periodontal disease-related phenotype (Socransky)
FHOD3	Periodontal disease-related phenotype (Socransky)
LINC02855, HAS2-AS1	Periodontal disease-related phenotype (Socransky)
HSP90AB2P, U6	Periodontal disease-related phenotype (Socransky)
GVINP1	Periodontal disease-related phenotype (Socransky)
AL356124.1	Periodontal disease-related phenotype (Socransky)
HSP90AB2P, U6	Periodontal disease-related phenotype (Socransky)
OSBPL10	Periodontal disease-related phenotype (Socransky)
AL355838.1	Periodontal disease-related phenotype (Socransky)

CDKL1	Periodontal disease-related phenotype (Socransky)
CRACR2A	Chronic periodontitis (localised)
KCNQ5	Periodontitis
GPR141, EPDR1	Periodontitis
C5AR1	Chronic periodontitis
DLG2	Chronic periodontitis
SIGLEC5, AC018755.2	Periodontitis
AP000959.1, MAPK6P2	Periodontitis
RNU6-675P, TEX51	Periodontitis
MIR297, LYPLA1P2	Periodontitis
AL109933.3	Periodontitis
LINC01239, AL391117.1	Periodontitis
AL354916.1, CUX2P1	Periodontitis
NUDT5	Periodontitis
AC004241.1	Periodontitis
HUNK	Periodontitis

Appendix S2. Genes and their corresponding proteins for stroke

Mapped gene	Reported trait
ZFHX3	Cardioembolic stroke (CCSp classification)
GNAO1	Cardioembolic stroke (CCSp classification)
LINC01438, PITX2	Cardioembolic stroke (CCSp classification)
ZFHX3	Cardioembolic stroke (TOAST classification)
LINC01438, PITX2	Cardioembolic stroke (TOAST classification)
ZFHX3	Cardioembolic stroke (CCS and TOAST classification)
PHF20	Cardioembolic stroke (CCS and TOAST classification)
LINC01438, PITX2	Cardioembolic stroke (CCS and TOAST classification)
ZFHX3	Cardioembolic stroke (CCS or TOAST classification)
LINC01438, PITX2	Cardioembolic stroke (CCS or TOAST classification)
ZFHX3	Cardioembolic stroke (CCSc classification)
AC010230.1, AC094104.2	Cardioembolic stroke (CCSc classification)
TTBK1, SLC22A7	Cardioembolic stroke (CCSc classification)
LINC01438, PITX2	Cardioembolic stroke (CCSc classification)
ATG7	Small vessel stroke
ATG7	Small vessel stroke
KNG1, AC068631.1	Small vessel stroke
FAT4, ANKRD50	Small vessel stroke
LINC02487, AL009178.1	Small vessel stroke
ATP6V1C1, MTND1P5	Small vessel stroke
DIO2-AS1	Small vessel stroke
SH2B3, ATXN2	Small vessel stroke (CCSp classification)
BRAP, ATXN2-AS	Small vessel stroke (CCSp classification)
CAMK2D	Small vessel stroke (CCS and TOAST classification)
SH2B3, ATXN2	Small vessel stroke (CCS or TOAST classification)
HDAC9, TWIST1	Large artery stroke (CCSp classification)j
AL049825.1, LINC01765	Large artery stroke (CCSp classification)j
HDAC9, TWIST1	Large artery stroke (TOAST classification)
LINC01492	Large artery stroke (TOAST classification)
HDAC9, TWIST1	Large artery stroke (CCS and TOAST classification)
AL049825.1, LINC01765	Large artery stroke (CCS and TOAST classification)
HDAC9, TWIST1	Large artery stroke (CCS or TOAST classification)
AL049825.1, LINC01765	Large artery stroke (CCS or TOAST classification)
AL109838.1, BTBD3	3-month functional outcome in lacunar ischaemic stroke (modified Rankin score)
AL133240.1, CPSF2	3-month functional outcome in lacunar ischaemic stroke (modified Rankin score)
SERPINA1	Large artery stroke
HDAC9	Large artery stroke
ACP1	Large artery stroke
AC007318.2, AC007318.3	Large artery stroke

SH3YL1	Large artery stroke
DTL	Large artery stroke
MCHR1	Large artery stroke
CARD9	Large artery stroke
ADAMTS2	Stroke (pediatric)
ADAMTS12	Stroke (pediatric)
TRIM29	Stroke (pediatric)
TRIM29	Stroke (pediatric)
PITX2, LINC01438	Stroke (ischemic)
IMPA2	Stroke
LINC00458, LINC00558	Stroke
CRYBG1	Stroke
RPL36AP26, AC006041.2	Stroke
PTPRG	Stroke
SPINK2	Stroke
TGFBI	Stroke
AL356094.2, AL356094.1	Stroke
LINC00709, AC044784.1	Stroke
WDFY4	Stroke
MICAL2	Stroke
ALDH1A2	Stroke
CHD3	Stroke
AP001347.1	Stroke
CLDN17, AF096876.1	Stroke
AL606519.1	Stroke
RNU6-1180P, IL1RN	Stroke
PTPRG	Stroke
LINC01411, SUMO2P6	Stroke
AC016573.1	Stroke
AL356094.2, AL356094.1	Stroke
HDAC9	Stroke
LINC00709, AC044784.1	Stroke
WDFY4	Stroke
MIR100HG	Stroke
AC018659.2, RMST	Stroke
AL356807.1, MTND4P33	Stroke
AL356807.1	Stroke
ALDH1A2	Stroke
AP001347.1	Stroke
CLDN17, AF096876.1	Stroke
NOS3	Stroke
COL4A1	Stroke
AP001407.1, DYRK1A	Stroke

LINC02455, NINJ2-AS1	Stroke
LINC02455, NINJ2-AS1	Stroke
LINC02455, NINJ2-AS1	Stroke
LINC02455, NINJ2-AS1	Stroke
AC244502.1	Cardiovascular death, myocardial infarction or stroke in response to clopidogrel treatment
RNU6-144P, ZYXP1	Cardiovascular death, myocardial infarction or stroke in response to clopidogrel treatment
PRKG1	Cardiovascular death, myocardial infarction or stroke in response to clopidogrel treatment
WFDC1	Cardiovascular death, myocardial infarction or stroke in response to clopidogrel treatment
LHFPL2	Cardiovascular death, myocardial infarction or stroke in response to clopidogrel treatment
MICAL2	Cardiovascular death, myocardial infarction or stroke in response to clopidogrel treatment
HDAC9	Stroke
HDAC9, TWIST1	Stroke (ischemic)
HDAC9, TWIST1	Stroke (ischemic)
LINC01438	Stroke (ischemic)
LINC01438	Stroke (ischemic)
ZFHX3	Stroke (ischemic)
SPSB4	Stroke (ischemic)
ASB3	Stroke (ischemic)
AC002996.1, ALDH2	Stroke (ischemic)
BSX, AP003040.1	Age-related diseases and mortality
AC125807.2, AC125807.1	Age-related diseases and mortality
GRHL1	Age-related diseases and mortality
AC079248.1, RNU7-2P	Age-related diseases and mortality
ITPK1	Age-related diseases and mortality
SEMA5B	Age-related diseases and mortality
ZFHX3	Age-related diseases and mortality
DNAH2	Age-related diseases and mortality
AC003986.1, AC007091.1	Age-related diseases and mortality
AKR1B1P6, THEMIS3P	Age-related diseases and mortality
CDKN2B-AS1	Age-related diseases and mortality
TMPRSS2, MX1	Age-related diseases and mortality
ANKRD26, FAM238C	Age-related diseases and mortality
TCF7L2	Age-related diseases and mortality
FADS1, FADS2	Age-related diseases, mortality and associated endophenotypes
ZPR1	Age-related diseases, mortality and associated endophenotypes
SIK3	Age-related diseases, mortality and associated endophenotypes

BSX, AP003040.1	Age-related diseases, mortality and associated endophenotypes
AC125807.2, AC125807.1	Age-related diseases, mortality and associated endophenotypes
ATP2B1	Age-related diseases, mortality and associated endophenotypes
ZCCHC8	Age-related diseases, mortality and associated endophenotypes
ITPK1	Age-related diseases, mortality and associated endophenotypes
ALDH1A2, LIPC, LIPC-AS1	Age-related diseases, mortality and associated endophenotypes
FTO	Age-related diseases, mortality and associated endophenotypes
HERPUD1, CETP	Age-related diseases, mortality and associated endophenotypes
ZFHX3	Age-related diseases, mortality and associated endophenotypes
AKR1B1P6, THEMIS3P	Age-related diseases, mortality and associated endophenotypes
SUGP1	Age-related diseases, mortality and associated endophenotypes
APOC1, APOC1P1	Age-related diseases, mortality and associated endophenotypes
AC243964.2, BCL3	Age-related diseases, mortality and associated endophenotypes
CELSR2	Age-related diseases, mortality and associated endophenotypes
GRHL1	Age-related diseases, mortality and associated endophenotypes
AC010872.2, APOB	Age-related diseases, mortality and associated endophenotypes
TDRD15, AC067959.1	Age-related diseases, mortality and associated endophenotypes
SLC30A3	Age-related diseases, mortality and associated endophenotypes
NRBP1, PPM1G	Age-related diseases, mortality and associated endophenotypes
GCKR	Age-related diseases, mortality and associated endophenotypes
AC079248.1, RNU7-2P	Age-related diseases, mortality and associated endophenotypes
ABCA12	Age-related diseases, mortality and associated endophenotypes
SEMA5B	Age-related diseases, mortality and associated endophenotypes
SLC39A8	Age-related diseases, mortality and associated endophenotypes
MIR297, LINC01438	Age-related diseases, mortality and associated endophenotypes
MAML3	Age-related diseases, mortality and associated endophenotypes

AC008897.2	Age-related diseases, mortality and associated endophenotypes
CERT1	Age-related diseases, mortality and associated endophenotypes
POLK	Age-related diseases, mortality and associated endophenotypes
POC5	Age-related diseases, mortality and associated endophenotypes
AC003986.1, AC007091.1	Age-related diseases, mortality and associated endophenotypes
BAZ1B	Age-related diseases, mortality and associated endophenotypes
VPS37D, MLXIPL	Age-related diseases, mortality and associated endophenotypes
AC100802.1, LPL	Age-related diseases, mortality and associated endophenotypes
AC091114.1, TRIB1	Age-related diseases, mortality and associated endophenotypes
CDKN2B-AS1	Age-related diseases, mortality and associated endophenotypes
PLCG1	Age-related diseases, mortality and associated endophenotypes
ADI1P1, ZHX3	Age-related diseases, mortality and associated endophenotypes
TMPRSS2, MX1	Age-related diseases, mortality and associated endophenotypes
ANKRD26, FAM238C	Age-related diseases, mortality and associated endophenotypes
TCF7L2	Age-related diseases, mortality and associated endophenotypes
LINC01394, AL034346.1	Stroke
MRPL58, RNU6-362P	Alzheimer's disease or small vessel stroke
FGA, FGB	Stroke
PDE3A	Stroke
CDKN2B-AS1	Stroke
SMARCA4	Stroke
CASZ1	Stroke
COL4A1	Stroke
LRCH1	Stroke
PMF1-BGLAP, PMF1	Stroke
AC010255.3	Stroke
WNT2B	Stroke
AC092720.1, AC092720.3	Stroke
ZFHX3	Stroke
LINC01438, PITX2	Stroke
TTBK1, SLC22A7	Stroke
BOLA3P1, RNU7-159P	Stroke
HDAC9, TWIST1	Stroke

ILF3	Stroke
SH3PXD2A, AL133355.1	Stroke
SH2B3, ATXN2	Stroke
AC017007.5, ANK2	Stroke
LINC02828	Stroke
CDK6	Stroke
LINC01394, AL034346.1	Stroke
ABO, AL772161.2	Stroke
FGA, FGB	Stroke
PMF1-BGLAP, PMF1	Stroke
PRPF8	Stroke
AC010255.3	Stroke
WNT2B	Stroke
RN7SL568P, CNOT6LP1	Stroke
AC092720.1, AC092720.3	Stroke
KCNK3	Stroke
ZFHX3	Stroke
LINC01438, PITX2	Stroke
TTBK1, SLC22A7	Stroke
BOLA3P1, RNU7-159P	Stroke
HDAC9, TWIST1	Stroke
ILF3	Stroke
SH3PXD2A, AL133355.1	Stroke
TSPOAP1-AS1, AC004687.2, RNF43	Stroke
SH2B3, ATXN2	Stroke
RPS10P21, AL139003.1	Stroke
AC017007.5, ANK2	Stroke
AC078880.1, AC008125.1	Stroke
CDK6	Stroke
RN7SL363P, FURIN	Stroke
LINC01394, AL034346.1	Stroke
HTRA1	Stroke
ABO, AL772161.2	Stroke
PTPRF	Stroke
HDAC9, TWIST1	Large artery stroke
CWF19L2, ALKBH8	Large artery stroke
LINC02113, GUSBP8	Large artery stroke
TTLL5	Large artery stroke
CDKN2B-AS1	Large artery stroke
AL136140.2, AL133334.1	Large artery stroke
TMEM63C, AC007375.2, ZDHHC22	Large artery stroke
RPL21P111, OBI1	Large artery stroke

TMEM163	Large artery stroke
CACNB2	Small vessel stroke
AC092720.1, AC092720.3	Small vessel stroke
CDKN2B-AS1	Coronary artery disease or large artery stroke
LPA	Coronary artery disease or large artery stroke
HDAC9, TWIST1	Coronary artery disease or large artery stroke
SMARCA4	Coronary artery disease or large artery stroke
PHACTR1	Coronary artery disease or large artery stroke
ATXN2-AS, BRAP	Coronary artery disease or large artery stroke
PSRC1, CELSR2	Coronary artery disease or large artery stroke
TCF21	Coronary artery disease or large artery stroke
EDNRA, PRMT5P1	Coronary artery disease or large artery stroke
PLPP3, AC119674.2	Coronary artery disease or large artery stroke
WDR12	Coronary artery disease or large artery stroke
AC020558.5, SMCR2	Coronary artery disease or large artery stroke
ZC3HC1, AC073320.2	Coronary artery disease or large artery stroke
ZPR1	Coronary artery disease or large artery stroke
CNNM2	Coronary artery disease or large artery stroke
AC022748.2, CHRNB4	Coronary artery disease or large artery stroke
ABO, AL772161.2	Coronary artery disease or large artery stroke
AC022335.1, SLCO1B1	Ischemic stroke (large artery atherosclerosis)
FAF1	Ischemic stroke (large artery atherosclerosis)
CDKN2C	Ischemic stroke (large artery atherosclerosis)
HDAC9	Large artery stroke
TOM1L1	Large artery stroke
AC113386.1	Large artery stroke
LINC02488, AC018754.1	Large artery stroke
RGS9, LINC02563	Large artery stroke
PARK7	Ischemic stroke
SHISA6	Ischemic stroke
NAA25	Ischemic stroke
LINC01438, PITX2	Ischemic stroke
AL034417.3	Ischemic stroke
SPSB4	Ischemic stroke
PHACTR1	Coronary artery disease or ischemic stroke
ZC3HC1, AC073320.2	Coronary artery disease or ischemic stroke
ABO, AL772161.2	Coronary artery disease or ischemic stroke
AC022748.3, AC022748.2	Coronary artery disease or ischemic stroke
PSRC1, CELSR2	Coronary artery disease or ischemic stroke
SMCR2, AC020558.5	Coronary artery disease or ischemic stroke
WDR12	Coronary artery disease or ischemic stroke
TCF21	Coronary artery disease or ischemic stroke
PLPP3, AC119674.2	Coronary artery disease or ischemic stroke

LINC01626, AL035467.2	Coronary artery disease or ischemic stroke
CDKN2B-AS1	Coronary artery disease or ischemic stroke
ATXN2-AS, BRAP	Coronary artery disease or ischemic stroke
LPA	Coronary artery disease or ischemic stroke
SMARCA4	Coronary artery disease or ischemic stroke
KNG1, AC068631.1	Thrombosis
F2	Thrombosis
LRAT, FGG	Thrombosis
F5	Thrombosis
FUNDC2	Thrombosis
SLC44A2	Thrombosis
PROCR	Thrombosis
COX7A2L	Thrombosis
TSPAN15	Thrombosis
AL357518.2, AL357518.1	Thrombosis
GAPDHP50, ICE2P2	Thrombosis
ABO	Thrombosis
F11-AS1	Thrombosis
AL136140.2, AL133334.1	Stroke (ischemic)
LINC01765, AL049825.1	Ischemic stroke (large artery atherosclerosis)
HDAC9	Ischemic stroke (large artery atherosclerosis)
F11-AS1	Ischemic stroke (cardioembolic)
CAV2	Ischemic stroke (cardioembolic)
ABO, AL772161.2	Ischemic stroke (cardioembolic)
AL023495.1	Ischemic stroke (cardioembolic)
BNIP1, RPL7AP33	Ischemic stroke (cardioembolic)
NEURL1	Ischemic stroke (cardioembolic)
HK1	Ischemic stroke (cardioembolic)
ZFHX3	Ischemic stroke (cardioembolic)
LINC01438, PITX2	Ischemic stroke (cardioembolic)
AC011029.1, HNF4G	Ischemic stroke (cardioembolic)
ZFHX3	Ischemic stroke (cardioembolic)
LINC01438, PITX2	Ischemic stroke (cardioembolic)
RGS7	Ischemic stroke (cardioembolic)
ABO, AL772161.2	Ischemic stroke (cardioembolic)
BNIP1, RPL7AP33	Ischemic stroke (cardioembolic)
NBEAL1	Ischemic stroke (small-vessel)
CAMK2D	Ischemic stroke (small-vessel)
COL4A2	Ischemic stroke (small-vessel)
CASZ1	Ischemic stroke (small-vessel)
PMF1-BGLAP, PMF1	Ischemic stroke (small-vessel)
AC010255.3	Ischemic stroke (small-vessel)
SH3PXD2A, AL133355.1	Ischemic stroke (small-vessel)

SH2B3, ATXN2	Ischemic stroke (small-vessel)
AC092720.1, AC092720.3	Ischemic stroke (small-vessel)
LINC01394, AL034346.1	Ischemic stroke (small-vessel)
AC092720.1, AC092720.3	Ischemic stroke (small-vessel)
LINC01492	Ischemic stroke (large artery atherosclerosis)
AL049825.1, LINC01765	Ischemic stroke (large artery atherosclerosis)
AC093908.1, EDNRA	Ischemic stroke (large artery atherosclerosis)
HDAC9, TWIST1	Ischemic stroke (large artery atherosclerosis)
MMP12, MMP3	Ischemic stroke (large artery atherosclerosis)
AC108751.2, AC108751.3	Ischemic stroke (large artery atherosclerosis)
LINC01492	Ischemic stroke (large artery atherosclerosis)
AL049825.1, LINC01765	Ischemic stroke (large artery atherosclerosis)
FARP1	Ischemic stroke (large artery atherosclerosis)
AC093908.1, EDNRA	Ischemic stroke (large artery atherosclerosis)
HDAC9, TWIST1	Ischemic stroke (large artery atherosclerosis)
SH2B3, ATXN2	Ischemic stroke (large artery atherosclerosis)
AL109933.2	Ischemic stroke (large artery atherosclerosis)
MMP12, MMP3	Ischemic stroke (large artery atherosclerosis)
CDKN2B-AS1	Stroke
SMARCA4	Stroke
CASZ1	Stroke
LRCH1	Stroke

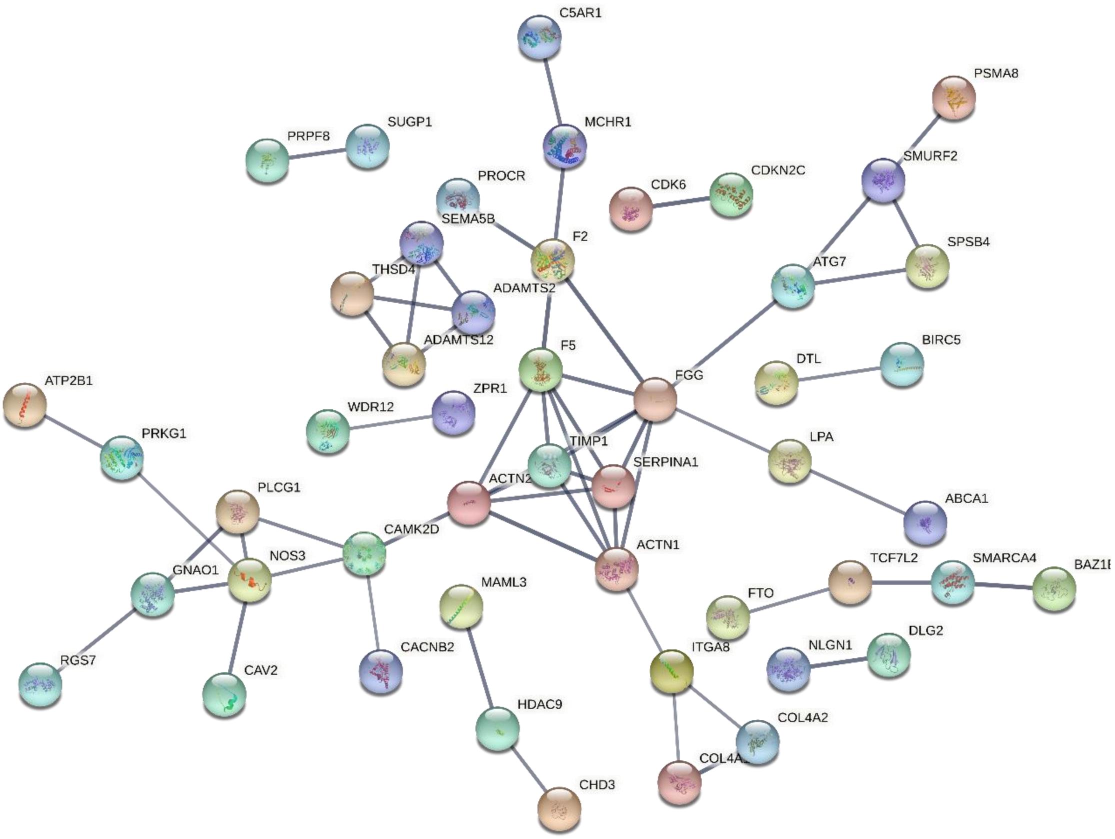

Appendix S3. PPI Network with confidence cut-off of 0.7.

number of nodes:	148
number of edges:	58
average node degree:	0.784
avg. local clustering coefficient:	0.243
expected number of edges:	41
PPI enrichment p-value:	0.00751

Appendix S4. PPI Network using Genemania tool.

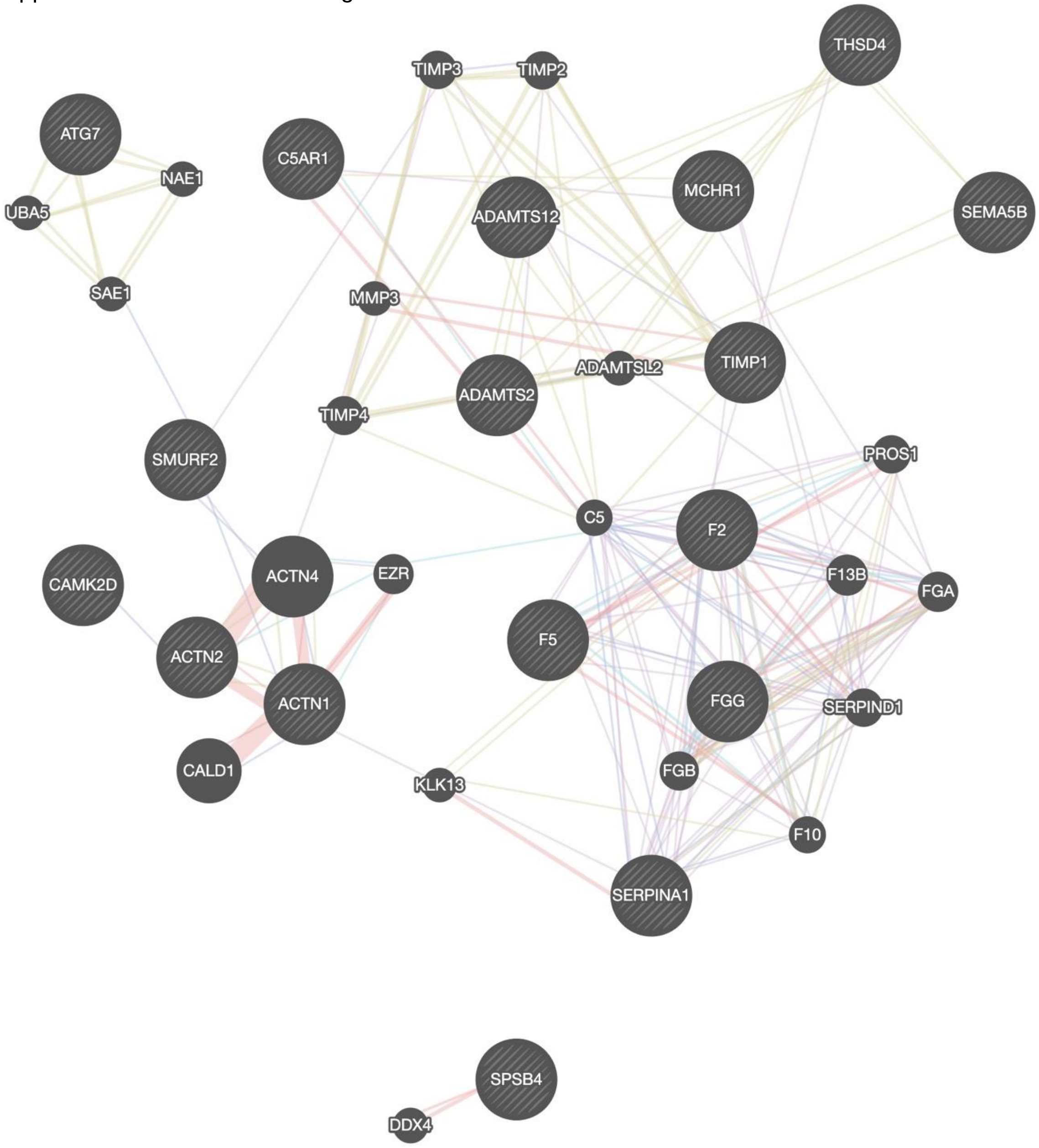

Appendix S5. Enrichment analysis

Pathway	Description	Count in Network	Strength	False Discovery Rate
HSA-140875	Common Pathway of Fibrin Clot Formation	4 of 22	1.38	0.0211
HSA-442729	CREB phosphorylation through the activation of CaMKII	3 of 18	1.34	0.0328
HSA-442982	Ras activation upon Ca2+ influx through NMDA receptor	3 of 20	1.3	0.0384
HSA-438066	Unblocking of NMDA receptors, glutamate binding and activation	3 of 20	1.3	0.0384
HSA-5083635	Defective B3GALTL causes Peters-plus syndrome (PpS)	4 of 37	1.16	0.0262
HSA-5173214	O-glycosylation of TSR domain-containing proteins	4 of 38	1.14	0.0262
HSA-114608	Platelet degranulation	7 of 125	0.87	0.0211
HSA-76002	Platelet activation, signaling and aggregation	8 of 256	0.62	0.0418
HSA-109582	Hemostasis	15 of 601	0.52	0.0211
